# Raising awareness about microbial antibiotic resistance in undergraduate dental students: a research-based strategy for teaching non-laboratory elements of a microbiology curriculum

**DOI:** 10.1186/s12909-020-1958-3

**Published:** 2020-02-11

**Authors:** Veronica Veses, Maria del Mar Jovani-Sancho, Raquel González-Martínez, Isidoro Cortell-Ballester, Chirag C. Sheth

**Affiliations:** 1 Department of Biomedical Sciences, Faculty of Health Sciences, Universidad Cardenal Herrera, CEU Universities, 46113 Valencia, Spain; 2 Department of Dentistry, Faculty of Health Sciences, Universidad Cardenal Herrera, CEU Universities, 46113 Valencia, Spain; 3 Department of Medicine, Faculty of Health Sciences, Universidad Cardenal Herrera, CEU Universities, Valencia, Spain

**Keywords:** Dental undergraduates, Research-based learning, Non-laboratory elements, Microbiology curriculum, Student learning objectives, Antimicrobial resistance

## Abstract

**Background:**

Resistance to antimicrobial agents has become a problem in modern society. Antibiotic resistant bacteria undermine the prevention and treatment of infections. Undergraduate dental students in Europe are required to receive information in aspects of microbiology relevant for dental practice, including oral microbial pathogens and resistance mechanisms against antimicrobial compounds. The objective of this study was to implement a research-based strategy to aid the understanding of the increase in antimicrobial resistance in undergraduate dental student training. The primary outcome of this project is the efficacious delivery of the learning objectives.

**Methods:**

Ten volunteer undergraduate student “ambassadors” were recruited to manage the project with assistance from lead academics. Student ambassadors were a source of peer learning for their colleagues. The project consisted of three phases: Pre-project preparation (in which the ambassadors received special instruction and training); Practical experience (in which the ambassadors worked with volunteer student colleagues to carry out the project); Public presentation of results (in which ambassadors presented study results at a scientific conference of their choosing).

**Results:**

A total of 1164 students volunteered for the project, corresponding to an average participation rate of 76.4% students per year of the course. Following final debriefing, student participants and ambassadors were strongly positive in their evaluation of the achievement of 8 key student learning objectives. The results demonstrate that most volunteers improved their knowledge related to antimicrobial resistance mechanisms in microbiology. Additional benefits of participation in this project included an improvement in dental knowledge and ethics in biomedical research for the student volunteers, whilst the student ambassadors reported improved knowledge about critical thinking and study design, as well as a deeper understanding about microbiological analysis methods.

**Conclusions:**

To the best of our knowledge, this the first instance of the application of project-based methodologies to the teaching of a traditionally non-laboratory component of a subject taught in the dentistry curriculum. Results from both students and ambassadors highlighted the increase in dental knowledge and an increased awareness of antimicrobial resistance as the key outcomes of this project.

## Background

Resistance to antimicrobial agents has grown from an emerging threat to a very real problem in modern society [1]. Bacteria resistant to multiple antibiotics (multidrug resistant organisms; MDRO) are being isolated with increasing frequency from hospitals, dental clinics and medical facilities around the world [2, 3]. Antimicrobial resistance undermines the successful prevention and treatment of a growing number of bacterial, parasitic and fungal infections, and it is driven by the misuse and overuse of antibiotics and poor infection prevention and control, amongst others [4].

In 2015, the World Health Assembly drew up a global plan to tackle this issue, establishing the crucial role of governments and the society as a whole [5]. The main focus of this plan includes surveillance in order to generate robust data, and implementation of antimicrobial stewardship programs across health care facilities [6]. In this context, the World Health Organization (WHO) has underlined the importance of undergraduate training in adequate prescribing practices [7].

The European Dental Undergraduate Curriculum is fairly uniform within the member countries of the European Union as it is regulated by the European Directive 78/687/EEC [8] Specifically, the Dentistry degree curriculum at Universidad Cardenal Herrera CEU requires the inclusion of aspects of microbiology relevant for dental practice, such as prevention of cross-contamination in the dental clinic, information regarding oral microbial pathogens and resistance mechanisms against antimicrobial compounds [8]. Therefore, the Dentistry Faculty plays a pivotal role in raising awareness amongst undergraduate dental students with regards to MDROs and their propagation in clinical settings.

Current guidelines about the teaching-learning processes in the higher education framework recommend the use of student-centered approaches, which combine problem-based, integrated educational philosophies [9]. Furthermore, newer generations of undergraduate students are demanding teaching practices that encourage student engagement and self-directed learning [10]. Strategies such as problem-based learning and learning by doing have been proven to increase motivation amongst students [10].

The objective of this study was to evaluate the implementation of a student-led, research-based strategy to teach non-laboratory elements of the microbiology curriculum for dentistry, specifically, the increase in antimicrobial resistance. The theoretical concepts relating to antimicrobial resistance are taught as part of the second year subject Microbiology and Virology (6 ECTS, first semester). Methicillin-resistant *Staphylococcus aureus* (MRSA) was selected as a model microorganism for this project for the following four reasons:

1. MRSA is a bacterium that can cause infections that are resistant to common antibiotics, such as penicillin, amoxicillin, and methicillin, and as such are associated with increased healthcare costs, longer hospital stays, and higher mortality [11].
2. Studies have shown that nasal colonization by MRSA in dentists and dental students is higher than in the general population [12–14].
3. The dental clinic constitutes an environment in which the transmission of MRSA, and potentially other multi-resistant microorganisms, is possible, due to the physical proximity with the patient and the possible contact with oral aerosols during treatment [3, 12]. MRSA has been isolated from dental impressions, gypsum casts [15] and dental impression guns [16].
4. Simple tools are easily available for the definitive isolation and identification of MRSA from non-invasive samples in a dental clinical setting.

## Methods

In this study, a small group of 10 student ambassadors, worked with academics to design and execute a research project involving student peer volunteers. The primary outcome was the degree of achievement of the established intended learning objectives (ILO) (Table 1), evaluated via a voluntary questionnaire. Student ambassadors investigated the presence or absence of MRSA in the nasal cavities of their colleagues.

**Table 1:** Intended learning objectives established for the evaluation of study outcomes

Intended Learning Objective	Principal Beneficiaries
Increased awareness of antimicrobial resistance	Student Ambassadors and Student Participants
Ethics and research	Student Ambassadors and Student Participants
Collaborative learning	Student Ambassadors and Student Participants
Improvement in dental knowledge	Student Ambassadors and Student Participants
Scientific communication	Student Ambassadors and Student Participants
Critical thinking and study design	Student Ambassadors
Microbiological analysis methods	Student Ambassadors
Interpersonal communication skills and leadership	Student Ambassadors

The approach consisted of a guided research project in which two undergraduate dental students from each of the five year groups would act as student ambassadors for the project. These students would then work together with the lead academics to facilitate the practical aspects of the research as well as act as intermediaries and as a source of peer learning for their colleagues. The project consisted of three clearly defined phases (the key beneficiaries of the phases are shown in parentheses):

1. Pre-project preparation (All undergraduate dental students)
  - *Basic training and recruitment of Student Ambassadors*
  - *Establishment of Intended Learning Objectives*
2. Practical experience (All undergraduate dental students)
  - *Project design*
  - *Identification of MRSA isolates in student samples*

### Statistical analysis

3. Public presentation of results (Student Ambassadors)

The three phases are described in greater detail below.

1. *Pre-project preparation*
  - *Basic training and recruitment of Student Ambassadors*

The initial phase of the project involved recruitment of students who would play the role of ambassadors when interacting with their colleagues. The student ambassadors’ responsibilities included participation in the practical elements of the project as well as serving as a source of peer information on antimicrobial resistance for their colleagues.

Prior to running the project, all students and ambassadors received a two-hour seminar in which the key theoretical and practical concepts related to the study were shared. The main concepts presented in the seminar session included microbial structure and function; prevalence and incidence of MRSA colonization and infection; transmission of MRSA in the dental setting and antimicrobial resistance mechanisms. The seminar contents were shared with the students in PDF format for future reference. The academics were specially chosen to vertically cover the entire degree program and ensure adoption of the knowledge from this experience by students in all 5 years of the curriculum. Specifically, seminars were delivered by the authors, to students as part of their training in the following subjects; Special Anatomy (1st year students, ICB); Microbiology and Virology (2nd year students, VV); Pathology and Therapeutic Dentistry (3rd year students, MMJS); Epidemiology and Public Health (4th year students, CCS) and Periodontics (5th year students, RGM).

Student ambassadors later met with the lead academics for a second briefing regarding the main objectives and protocol for the project as well as explaining the learning objectives and the implications of their role (the level of knowledge of the student ambassadors was informally confirmed through discussion sessions with the academics). The academics encouraged the ambassadors to supplement the material by searching for current literature via standard scientific literature search engines, such as PubMed, ScienceDirect and Google Scholar. Weekly meetings followed the initial phase of the project.

b. *Establishment of Intended Learning Objectives*

The student learning objectives shown in Table 1 were established following consultation with the academics implementing the project, and in concordance with the overall learning objectives.

2. Practical Experience

Two student ambassadors were selected from each year of the degree program (total 5 years) so that students in all groups always had access to an informed peer. A total of 10 student ambassadors were recruited for this project. The student ambassadors were responsible for providing peer support to their colleagues and obtaining informed consent for the obtention and processing of samples.

- Project design

The project followed a cross-sectional design and was performed in agreement with the STROBE guidelines for observational studies [17]. The participants were undergraduate dental students of the Universidad Cardenal Herrera CEU (Valencia, Spain) recruited by the student ambassadors under the guidance of the lead academics, between September 2015 and July 2017. The study was approved by the Ethics Committee of CEU Cardenal Herrera University (authorization number CEI15/003).

b. *Identification of MRSA isolates in environmental and individual samples*

Student ambassadors were responsible for all sampling under the supervision of the lead academics. Both nares of undergraduate dental students who had consented to participate were sampled with a sterile pre-moistened cotton swab, as described elsewhere [14]. Sample collection was performed during January and February of 2016 and 2017, corresponding to the cold season in Spain, to avoid the effects of seasonality in the prevalence data [18]. All samples obtained were streaked immediately on chromogenic ChromID MRSA (BioMerieux) and incubated at 37 °C for 24 h. Positive MRSA colonies were manually counted as described previously [19], and colonization levels of the participants were recorded. The lead academics employed the Staphytect Plus Kit (Oxoid, UK) to confirm the isolates as *S. aureus*, following manufacturers’ instructions [20].

c. Data collection and Statistical analysis

Two questionnaires (online cross-sectional surveys) for evaluating the student learning objectives (one for participants and the other for student ambassadors) were designed bespoke for this project in order to describe the degree of achievement of the established student learning outcomes. The student participant questionnaire consisted of 5 questions, one for each student objective considered, whilst the ambassador questionnaire consisted of 8 questions, reflecting the increased responsibility and expected learning outcomes for this group of students. Participants answered the questions by selecting a response based on a 5 point Likert scale (Table 2). Both tools were validated for legibility and comprehension amongst a cohort of student peers not involved in the research project. The questionnaires were built and hosted on Microsoft Office 365: Forms and a link was provided to students following the study in order to collect their opinions anonymously. Descriptive statistical analysis was carried out on the survey data, with the results being presented as frequency counts and percentages, calculated using Microsoft Excel.

3. Public presentation of results

**Table 2:** Questionnaires for evaluating the intended learning objectives amongst student participants and ambassadors

Intended Learning Objective	Student Questionnaire	Ambassador Questionnaire
Increased awareness of antimicrobial resistance	1) To what degree did your participation in the MRSA project increase your awareness of antimicrobial resistance? • Not at all • Not so well • Somewhat well • Well • Extremely well	
Ethics and research	2) To what degree did your participation in the MRSA project increase your awareness of ethics and research? • Not at all • Not so well • Somewhat well • Well • Extremely well	
Collaborative learning	3) Did you find that the project encouraged you to collaborate more with your peers? To what degree? • Not at all • Not so well • Somewhat well • Well • Extremely well	
Improvement in dental knowledge	4) Did you find that the project improved your dental knowledge in general? To what degree? • Not at all • Not so well • Somewhat well • Well • Extremely well	
Scientific communication	5) What impact did your participation in the MRSA project have on your scientific communication skills? • None at all • Not much • Somewhat • Positive • Extremely positive	
Critical thinking and study design		6) Did you feel that your participation in the MRSA project helped develop your critical thinking and study design skills? To what extent? • None at all • Not much • Somewhat • Positive • Extremely positive
Microbiological analysis methods		7) To what extent did you feel that your participation in the MRSA project helped you increase your knowledge of microbiological analysis methods? • None at all • Not much • Somewhat • Positive • Extremely positive
Interpersonal communication skills and leadership		8) Do you feel that you have improved your interpersonal communication and leadership skills as a result of your participation in this project? • Not at all • Not so well • Somewhat well • Well • Extremely well

The student ambassadors, in conjunction with volunteers from amongst their colleagues were tasked with the responsibility of identifying a suitable conference for the presentation of the results of the study. The lead academics of the project worked together with the ambassadors and student volunteers to register for the conference and in the preparation of the presentation material.

## Results

### Student participation

The main objective of this study was to evaluate the implementation of a student-led, research-based strategy to teach non-laboratory elements of the microbiology curriculum for dentistry, specifically, the increase in antimicrobial resistance. The project was carried out within the context of the second-year undergraduate subject, Microbiology and Virology, which is mandatory for all undergraduate students. Table 3 shows the proportion of students enrolled in each year of the degree program, along with the proportion of students who chose to participate in the project. The student ambassadors are counted amongst the participating students as they received the theoretical material and submitted a sample for analysis.

**Table 3:** Number of students per year of the degree program, with the proportion choosing to participate in the project

Degree year	Total number of students invited to participate	Number of participating students	Proportion of participating students (%)
1st	375	254	67.7
2nd	342	242	70.8
3rd	264	224	84.8
4th	275	226	82.2
5th	285	218	76.5
Mean	308.2	232.8	76.4

The mean participation was 232.8 students per year of the degree program, which corresponds to 76.4% of the students per year, on average.

### Achievement of intended learning objectives

**Fig. 1:**
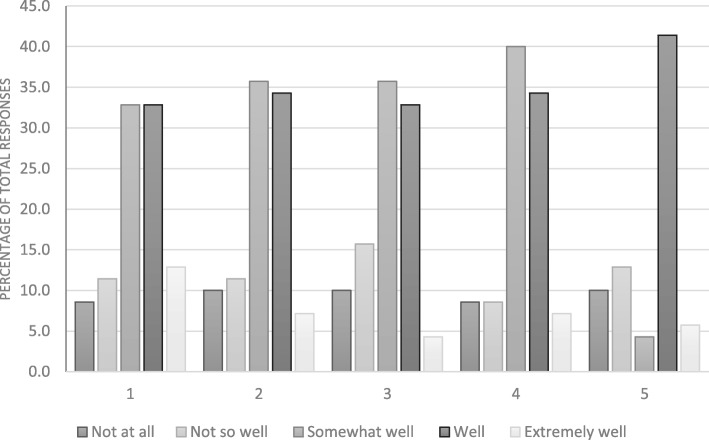
Distribution of responses to the student evaluation questionnaire, grouped by question

**Fig. 2:**
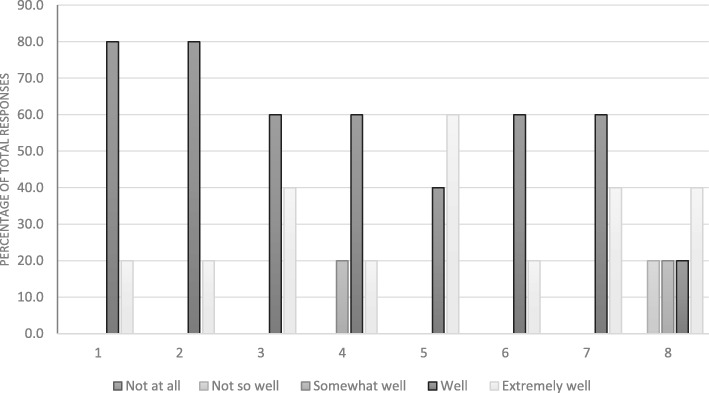
Distribution of responses to the student ambassador evaluation questionnaire, grouped by question

The overall response rate amongst student volunteers was 27.5%, whilst 50% of the ambassadors responded to the evaluation questionnaires. An analysis of the responses to the questionnaires are displayed in Fig. 1 (student volunteers) and Fig. 2 (student ambassadors).

Our analysis shows that student participants responded most positively (combining somewhat well, well, and extremely well) toward intended learning objectives 1 and 4 (78.6 and 81.4% respectively), corresponding to questions regarding their increased awareness of antimicrobial resistance and improvement in dental knowledge respectively (Fig. 1). This was followed by their self-reported acquisition of ILO 3 (77.1%, improvement of knowledge regarding ethics and research practices). 72.9% of student participants responded positively towards the collaborative learning aspects of this project whilst only 51.4% of students felt that the study aided improved their scientific communication skills (Fig. 1). Global analysis of the complete questionnaire revealed that 72.3% of the questions were answered positively (somewhat well, well or extremely well), whilst 21.4% of responses were negative (not so well or not at all).

Amongst the student ambassadors, the results in general were more positive than the student volunteers (Fig. 2). The evaluation questionnaires for the student ambassadors contained three additional questions (Table 2, questions 6–8), reflecting their increased responsibility and involvement in the project. In contrast to the student volunteers, the ambassadors responded overwhelmingly positively (100% of responses were somewhat well, well or extremely well) to questions 1–5 (Fig. 2). When asked about the impact of the project on ambassadors critical thinking skills, 20% responded “extremely well”, whilst 60% responded “well”. 100% of ambassadors felt that the project allowed them to increase their knowledge in microbiological research methods (40% responded “extremely well”; 60% responded “well”). The evaluation of the impact of the project on the development of interpersonal skills and leadership ability amongst the ambassadors was more varied (Fig. 2).

### Public presentation of results

The student ambassadors identified the Congreso Internacional de Estudiantes (“International Student Congress”; CIE13) in 2016, hosted by the CEU Cardenal Herrera University as a target for the presentation of the results of the project. A cohort of students were selected for an oral presentation, titled “Study of the prevalence of Methicillin-resistant *Staphylococcus aureus* in UCHCEU Dentistry Students”. Their presentation was awarded first prize in the undergraduate student research category.

### Anonymity and other special considerations

Taking into consideration the social and psychological effects of a student discovering they (or one of their colleagues) are a carrier of nasal MRSA, the analysis was anonymized, and student ambassadors were not aware of the identity of student colleagues with positive results. Lead academics contacted colonized students privately and during one-to-one meetings, explained the results and implications of the study, and answered all questions arising.

All asymptomatic carriers were offered decolonizing treatment using nasal mupirocin, twice a day for five days, as indicated by national guidelines [21]. A second nasal swab was obtained 15 days after the completion of the treatment to confirm the eradication of MRSA. Two students developed higher levels of colonization following the decolonization treatment. Fusidic acid was prescribed to these students, twice a day for five days, as specified by national guidelines as a secondary treatment following the failure of the mupirocin therapy [21]. All students were declared MRSA-free by the end of the study.

## Discussion

The development of creative, engaging programs to stimulate learning of key, contemporary issues in modern dentistry programs is of the highest importance. Multiple authors describe the benefits of student engagement in self-driven research projects with key outcome goals as a strategy to enhance the learning process [22–24]. The Microbiology and Virology curriculum is particularly susceptible to such enhancement as students traditionally find the subject complex, dry and difficult to apply to their activity as a dentist. In this article, we describe an innovative project, designed to enhance the understanding of a key concept in undergraduate dentistry; that of the resistance to antimicrobial compounds. The project, led by student ambassadors, in a collaborative peer-learning environment used effective, easy to perform tests for the detection of antibiotic resistant bacteria (MRSA) in the nasal fossae of student colleagues.

The achievement of intended learning outcomes was evaluated by anonymous electronic questionnaires. The evaluation methodology as described in our study are reflected in similar projects employing inquiry- or project-based learning strategies [24, 25]. The introduction of project-based learning in microbiology curriculum reform enhanced the consolidation of theory knowledge, improved experimental skills, cultivated innovative scientific thinking, and strengthened the awareness of oral health in a study from Japan [26]. In another study, this time focusing on teaching cell biology to dental students, the authors found that students receiving the project-based learning approach were more familiar with the importance methodological issues, were able to produce written reports of higher quality, better understood the relationship between their subject and the practice of dentistry and were better able to recognize the relevance of biomedical investigation as compared to control students receiving the traditional lecture—based approach [23]. There are few standardized, well designed controlled trials investigating the effectiveness of problem- or project-based learning in dentistry [27] and, to the best of our knowledge, this is the first study of its kind to investigate the outcomes of project-based learning on non-laboratory-skills elements of microbiology teaching.

The application of project-based learning to university curricula is not a new phenomenon [22, 28]. One of the keys to success in this type of project is student involvement and uptake [29, 30]. The elevated participation rates (76%) amongst dental students in this study, indicated that the majority of students were sufficiently interested in participating. The voluntary nature of the participation in the study was especially important as we were able to work with students who were interested and invested in participating to ensure the success of the program. It could be reasonable to expect that the analysis of the intended learning outcomes may decrease significantly in cases where this type of project would be imposed on the students, as seen elsewhere [31].

Our results demonstrate that the majority of student volunteers and all of the student ambassadors were able to achieve the primary objective, an increased student awareness of antimicrobial resistance. This work is supported by other studies showing that a research-based strategy may successfully be used to communicate theoretical components of the curriculum. A blended learning study by Van Dam et al. showed similar results in a graduate course in healthcare redesign [32], whilst de Lencastre *et. al.* demonstrated efficacy of the research-based approach in a graduate course for teaching modern DNA techniques [33].

The strengths of this study include the large number of participants and the structured, peer-led, research-focused approach applied to teaching antimicrobial resistance as part of the Microbiology and Virology course in a dentistry undergraduate curriculum. Student participation was voluntary, resulting in enhanced adoption and appreciation of the project. The voluntary nature of the feedback resulted in a lower than expected return rate for the questionnaires. This has been reported by other authors [34, 35] suggesting that low response rates may be due to survey fatigue experienced by undergraduate university students. In addition, upon reflection, we noted that the evaluation questionnaire was delivered to the students and ambassadors towards the end of an academic year and may have coincided with multiple deadlines thereby reducing the response rates. This could be remedied in future projects by changing the delivery format of the questionnaires from electronic to a system whereby students would respond to a paper copy of the questionnaire in a classroom setting followed by anonymous submission through a drop-box. An improvement in response rates could be expected by avoiding the overlapping of the evaluation phase of future projects with end of year deadlines. The development of scientific communication skills was one of the intended learning outcomes that received the lowest score amongst student volunteers. The fact that only the ambassadors and a small number of student volunteers presented the project in a conference may have contributed to this evaluation. In the future, it may be interesting to create multiple presentation opportunities for student participants, in conjunction with student ambassadors in order to share the experience more broadly amongst the participants.

## Conclusions

This study demonstrates the benefits of applying a project-based strategy to raising awareness about antimicrobial resistance as part of a course on microbiology and virology delivered to second year undergraduate dentistry students. Both student volunteers and ambassadors gave positive feedback related to an improvement in their dental knowledge and self-reported an increased awareness of antimicrobial resistance issues (the key objective of this study). Furthermore, student ambassadors developed a valuable understanding of microbiological research methods. To the best of our knowledge, this the first instance of the application of project-based methodologies to the teaching of a traditionally non-laboratory component of a subject taught in the dentistry curriculum.

## Data Availability

The datasets used and/or analyzed during the current study are available from the corresponding author on reasonable request.
